# Evaluating the myopia progression control efficacy of defocus incorporated multiple segments (DIMS) lenses and Apollo progressive addition spectacle lenses (PALs) in 6- to 12-year-old children: study protocol for a prospective, multicenter, randomized controlled trial

**DOI:** 10.1186/s13063-020-4095-8

**Published:** 2020-03-19

**Authors:** Yan Li, Yafei Fu, Kai Wang, Zhiming Liu, Xiaoqing Shi, Mingwei Zhao

**Affiliations:** 1 Department of Ophthalmology, Peking University People’s Hospital, Beijing, China; 2 Eye Diseases and Optometry Medical Research Institute, Peking University People’s Hospital, Beijing, China; 3 Beijing Key Laboratory of Diagnosis and Therapy of Retinal and Choroid Diseases, Beijing, China; 4 College of Optometry, Peking University Health Science Center, Beijing, China

**Keywords:** Myopia, Progression, Prospective, Multicenter, Trial, Myopic defocus, Spectacle lens

## Abstract

**Background:**

Myopia is increasing in prevalence and is currently recognized as a significant public health issue worldwide, particularly in China. Once myopia develops, appropriate clinical interventions need to be prescribed to slow its progression. Currently, several publications indicate that myopic defocus (MD) retards eye growth and myopia progression. However, no clinical trials have compared the outcomes of different MD spectacle lenses in the same observational group, especially in mainland China. The aim of the present study is to compare the myopia control efficiency of two different MD spectacle lenses: defocus incorporated multiple segments (DIMS) lenses and Apollo progressive addition lenses (PALs).

**Methods:**

The trial is designed as a 3-year, prospective, randomized, multicenter clinical trial of schoolchildren treated with DIMS lenses and PALs. A total of 600 Chinese primary school children aged 6–12 years will be recruited, and each group is intended to include 300 subjects. The inclusion criteria are myopia between − 1.00 and − 5.00 diopters and astigmatism ≤ 1.50 diopters. The follow-up time points will be 1 month (m), 3 m, 6 m, 12 m, 18 m, 24 m, 30 m, and 36 m. The primary outcome will be determined by the difference between the two groups in cycloplegic spherical equivalent refraction between baseline and the last follow-up visit. The secondary outcome is the axial length, and the exploratory outcomes include ocular biometric measures, peripheral refraction, binocular vision, accommodation, compliance, and the results of questionnaires related to wearing experiences.

**Discussion:**

The present study will be the first randomized controlled trial in myopic primary school children treated with DIMS lenses and PALs in China. The results will indicate whether and how much different MD mechanisms retard myopia progression and axial elongation. In addition, the comparison will provide information on the clinical efficacy and safety of DIMS lenses and PALs, including information related to wearing experiences and visual function.

**Trial registration:**

Chinese Clinical Trial Registry (ChiCTR), ChiCTR1900025645. Registered on 3 September 2019. http://www.chictr.org.cn/showproj.aspx?proj=42927.

## Background

It is estimated that myopia (also called “nearsightedness”) will affect 50% of the world population by 2050 [1–3]. With the growing prevalence of myopia in young generations, this “epidemic” disease is currently recognized as a public health issue, particularly in China [4]. The annual incidence of myopia onset between the ages of 7 and 15 years is constantly growing, and by the age of 18 years, ~ 80% of the urban-based Han population in mainland China is myopic, regardless of geographic locality. Controlling the progression of myopia and preventing complications of myopia that result in irreversible visual loss, such as myopia maculopathy, retinal detachment, glaucoma, and cataracts [5], will require collaborative efforts worldwide.

Several clinical interventions are currently used for myopia control, including spectacle lenses, contact lenses, and pharmacological treatments [6]. Regardless of the treatment strategy, slowing the progression of myopia after onset is the most important therapeutic goal [1, 4]. It has been reported that single-vision (SV) spectacle lenses designed to alter peripheral defocus achieve less than a 14% reduction in myopia progression. Bifocal vision and progressive addition spectacle lenses have shown variable clinically significant therapeutic effects between 6% and 50% compared with SV spectacles in different studies [7, 8]. Orthokeratology has proven to be effective in slowing myopia progression and axial elongation by between 30% and 55% [7]. Additionally, 0.01% atropine has shown an effect on refractive error retardation (~ 45%) and no apparent effect on axial length compared with historical control groups [7, 8].

Once a child has been diagnosed with myopia, an appropriate management strategy should be applied. In addition, several other aspects should be taken into account, such as age of onset, baseline refractive status, visual environment, compliance, risks and benefits of the treatment strategy, parental myopic status, and annual cost [7, 8]. Among all the treatment options, intervention with spectacle lenses is a simple as well as the least invasive method, in contrast to contact lenses and pharmacological treatments, for children and their parents, especially for children under 8 years old [7]. Considering numerous patient-specific factors related to myopia development and progression, the optimum prescription needs to be verified according to associated risk factors [4, 7].

Currently, there are several publications from animal and human studies showing that myopic defocus (MD) retards eye growth and myopia progression, while hyperopic defocus promotes eye growth compared with SV spectacle lenses [9–12]. In clinical, there are two major spectacle lenses designs based on the idea of MD: defocus incorporated multiple segments (DIMS) lenses and Apollo progressive addition lenses (PALs) (Apollo Eyewear, River Grove, IL, USA) [10, 13, 14]. Both are recommended to manipulate optical defocus across the visual field, which has been suggested to result in greater myopia control. However, to date, several issues remain under exploration, including (1) the efficacy of myopia control associated with added powers investigated systematically in the same observational, multicenter clinical trial; (2) the efficacy in 6- to 12-year-old primary school students in mainland China, who are especially prone to myopia progression; and (3) the quality of vision, which refers to the comfort and frequency of visual symptoms after wearing added-power spectacle lenses and is evaluated through questionnaires.

## Methods/design

### Aim of the study

The current prospective, multicenter randomized controlled trial will evaluate the myopia progression control efficacy of two broadly used clinical MD spectacle lenses (DIMS lenses and PALs) in 6- to 12-year-old myopic children in primary school. The primary aim is to determine whether DIMS lenses are noninferior to PALs in the combined endpoint of spherical equivalent refraction (SER) and axial length (AL) progression in myopic subjects over 3 years. Other changes will also be compared over the study period, including risk factors, ocular health, uncorrected relative peripheral refraction, binocular vision function (principally vergence), accommodation (particularly lag and amplitude), subfoveal choroidal thickness, visual environment, and wearing experiences [15].

### Study settings and responsibilities

Five trial sites will be involved, including Peking University People’s Hospital (PKUPH; Dr. Zhao Mingwei, principal investigator [PI]; Dr. Li Yan, co-PI), Peking University International Hospital (Dr. Li Mingwu, sub-PI), Kunming City Maternal and Child Health Hospital (Dr. Li Na, sub-PI), Beijing Haidian Maternal and Child Health Hospital (Dr. Chen Wei, sub-PI), and ChuiYang Liu Hospital affiliated with Tsinghua University (Dr. Wang Hongxing, sub-PI). Each of the hospitals is a large center with ophthalmology clinics and optometrists, and data will be collected at each site. The primary investigators at each site compose the steering committee, which is under the leadership of PKUPH (Dr. Zhao Mingwei, PKUPH, lead PI and chair). The steering committee will provide final approval of the protocol and any changes to the procedure during the clinical trial.

Among these sites, PKUPH will be in charge of supervising the conduct of the study, including staff training and assessment, protocol decisions and amendments, form development, data management, data analyses, and quality control. The reason for endowing PKUPH with this governance authority is that PKUPH has undertaken dozens of domestic and foreign multicenter clinical trials and thus has abundant clinical trial experience. In addition, PKUPH has a group of staff members who undertake site management organization (SMO) work, which will ensure that the clinical trial protocol will be strictly implemented at different study sites.

Coordinating responsibilities, such as data collection and recording, will be performed at all of the study sites. Subjects will be screened at each site to achieve a minimum screening percentage of 10% and a maximum percentage not exceeding 30%. All of the centers will continue to screen subjects until the target population is achieved.

### Study design and recruitment

This is designed as a 3-year, prospective, randomized, multicenter clinical trial. Recruitment is intended to begin on 30 October 2019 and is scheduled to end on 30 October 2020. A total of 600 primary school children (aged 6–12 years) will be recruited, and each of the participants will be followed for 3 years. Randomization will be performed with a random number table, and each group (DIMS or PALs) will contain 300 subjects. The final distance prescription will be determined by a masked investigator (MI) using the cycloplegic subjective refraction measured by phoropter after the objective refraction is measured by autorefraction. The lenses will be replaced with an updated prescription when the change in SER is greater than 0.50 diopters.

Potential participants will be recruited for the clinical trials at each center through two primary processes: (1) ophthalmologist referral during daily eye disorder treatment in outpatient clinics and (2) optometrist referral from optometry clinics during myopia treatment.

All of the potentially eligible participants and their parents/guardians will be contacted by a research coordinator who will explain the study in detail to ensure that the children and their parents/guardians understand the entire clinical trial. Once they are interested, both the patients and their parents/guardians will be seen in the clinical research laboratories to sign an informed consent form. Interested participants will be invited for the eligibility and baseline assessments by the study staff. All the identifying information will be confidential. It is estimated that ten new subjects will be recruited each month, on average, at each site.

### Myopia defocus spectacle lens systems and spectacle prescriptions

#### DIMS lenses

The DIMS lenses are custom-made plastic spectacle lenses. Each lens comprises a central optical zone (9 mm in diameter) for correcting distance refractive errors and an annular multiple focal zone with multiple segments (33 mm in diameter) having a relative positive power (+ 3.50 diopters). The diameter of each segment is 1.03 mm [10].

#### Apollo PALs

The Apollo PALs comprise an asymmetrical MD design with a 3 MD zone, including a + 2.50 diopters full positive power superior zone, an 80% full MD power nasal zone, and a 60% full MD power temporal zone.

Both of the spectacle lenses are designed to simultaneously provide clear distance vision for the wearer and introduce MD for the peripheral retina by providing a plane in front of the retina, resulting in signals being received as blurred images on the retina [5]. All of the children will be instructed to wear lenses all the time throughout the whole study. The use of atropine eye drops of any concentration will not be permitted for any participant during the study.

The final distance prescription of the spectacles will be determined on the basis of cycloplegic subjective refraction by the masked optometrist. The spectacle lenses will be replaced and upgraded when the change in SER is 0.5 diopters or more in either eye compared with refraction while wearing spectacles.

At the initial spectacle dispensing and at each follow-up visit, subjects and their guardians will receive face-to-face instruction about the purpose, use, and care of the lenses. In addition, investigators will explain the etiology and pathology of myopia, emphasize the importance of adherence to the follow-up protocol for evaluating myopia progression, and provide notifications regarding the follow-up ophthalmic examinations. In addition, the SMO from PKUPH will provide phone call reminders before each visit to enhance compliance with the present clinical trial.

### Eligibility criteria

The following eligibility criteria for this trial were modified from those provided by the International Myopia Institute (IMI) and related studies [6, 10, 15]:

- Mainland Chinese, Han nationality
- Age at enrollment: 6–12 years old
- Cycloplegic SER: − 1.00 to − 4.00 diopters, with SER calculated as the sphere plus 0.5 times the cylinder in diopters (Recommended dosage for cycloplegic refraction is two drops of 1% tropicamide given 5 min separately. Cycloplegic refraction outcomes should be measured 30–45 min after the first drop of tropicamide is instilled, which ensures the maximal cycloplegic effect.)
- Astigmatism: 1.50 diopters or less
- Anisometropia: 1.50 diopters or less
- Difference between the right and left pupil sizes: 2 mm or less
- Monocular best-corrected visual acuity (BCVA): 20/20 (0.0 logMAR) or better (logMAR chart)
- Willingness to wear spectacle lenses regularly
- Acceptance of random group allocation and the masked study design

The exclusion criteria are as follows:

- Strabismus: checked by cover test at far and near distances
- Any ocular and systemic diseases, including abnormalities, that might affect visual functions or refractive development
- Previous experience with myopia control, including orthokeratology, progressive addition spectacle lenses, bifocal lenses, and pharmaceutical treatment (e.g., atropine)

### Study outcomes and follow-up schedule

#### Rationale for outcome chosen

**Fig. 1:**
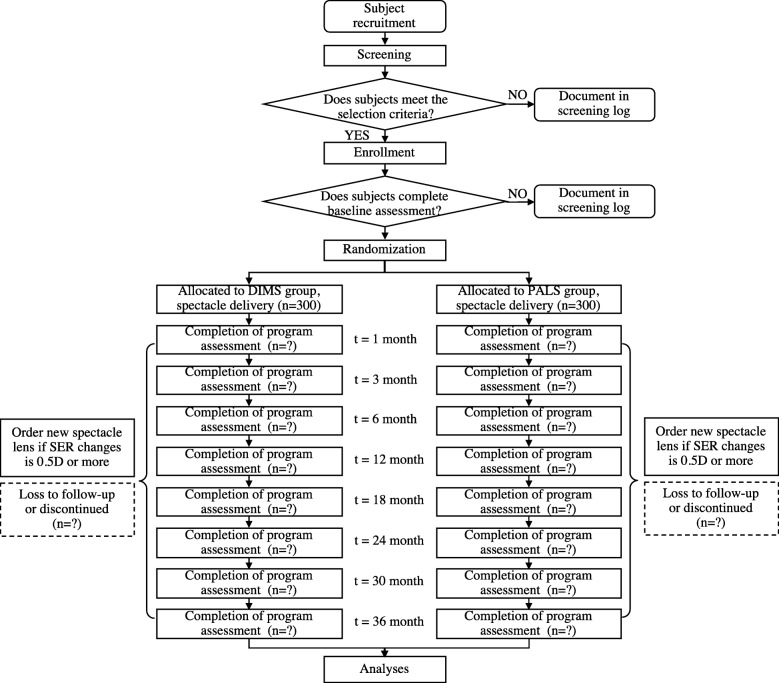
Schematic of the trial design

Myopia is an eye disorder in which light focuses in front of the retina, but not right on the retina, and mostly because of the excessive axial elongation of the eyeball. In the clinic, two valid and reproducible indicators—subjective refraction (SER in diopters) and AL (mm) under cycloplegia—are considered to be more relevant for evaluating changes in subjects with myopia. In addition, several influencing factors are related to the evaluation of myopia. Thus, in the present study, primary, secondary, and exploratory outcomes will be evaluated during the follow-up period according to the schedule (Fig. 1 and Table 1).

**Table 1:** Schedule of assessments and examination items

Procedures/measurements		Enrollment (− 2 to 0 week)	Baseline	1 Week (± 1 day)	1 Month (± 3 days)	3 Months (± 7 days)	6 Months (± 14 days)	12 Months (± 21 days)	18 Months (± 28 days)	24 Months (± 35 days)	30 Months (± 42 days)	36 Months (± 60 days)
Consent form signed			x									
Basic information	Demographics	x										
	History	x	x	x	x	x	x	x	x	x	x	x
Refraction	Noncycloplegic autorefraction	x	x				x	x	x	x	x	x
	Subjective refraction	x	x				x	x	x	x	x	x
	Cycloplegic subjective refraction^a^		x				x	x	x	x	x	x
	Cycloplegic autorefraction^a^		x				x	x	x	x	x	x
	Peripheral refraction^a^		x				x	x	x	x	x	x
Visual acuity	Habitual spectacle visual acuity	x	x				x	x	x	x	x	x
	Best corrected visual acuity	x	x				x	x	x	x	x	x
Ocular alignment	Cover test (distance, near)	x										
	Phoria (distance, near)		x				x	x	x	x	x	x
Accommodation	Lag		x				x	x	x	x	x	x
	Amplitude		x				x	x	x	x	x	x
Eye examinations	Slit-lamp examination, external ocular health check	x	x				x	x	x	x	x	x
	IOP measurement	x	x				x	x	x	x	x	x
	Pupil size		x				x	x	x	x	x	x
	Keratometry		x				x	x	x	x	x	x
	Contrast sensitivity											
	Stereopsis		x				x	x	x	x	x	x
	Axial length (mm)^a^ (IOLMaster [Zeiss, Oberkochen, Germany], measure between 9:00 and 11:00 a.m.)		x				x	x	x	x	x	x
	Choroidal thickness measurement		x				x	x	x	x	x	x
	Fundus examination^a^		x				x	x	x	x	x	x
Questionnaire	Visual habits		x									x
	Spectacle lens performance		x									x

*IOP* intraocular pressure

^a^With cycloplegia

#### Primary outcome

The primary outcome is to determine whether DIMS lenses are noninferior to PALs for the prevention of myopia progression by evaluating the cycloplegic SER changes in two groups of subjects. For the primary outcome analyses, myopia progression over 3 years will be determined by the difference in the subjective SER between baseline and the last follow-up visit. Other measurements obtained at follow-up visits are considered secondary outcome measures.

Procedures for subjective refraction will be as follows [10]:

1. Starting with autorefraction and refine subjectively
2. Occlude the left eye
3. Determine best sphere first
4. Determine cylindrical error
5. Refine sphere using + 1.00 diopter blur back test monocularly and finish by offering a binocular + 0.25 diopters additional
6. Binocular balance (prism dissociation); stop when no difference between the upper and lower line

#### Secondary outcome

The secondary outcome is to determine whether DIMS lenses are noninferior to PALs for the prevention of axial elongation (mm) determined by noncontact interferometry measurements [10] in the two groups of subjects at baseline and the last follow-up visit.

#### Exploratory outcomes

Several innate and environmental factors are useful in understanding the control of myopia progression [6, 8, 15], including age and refractive error at onset, family history (e.g., parental myopic status), visual and environmental habits (e.g., near work time, outdoor time, spectacle wear time, brightness of light exposure), binocular vision (e.g., accommodative lag, elevated accommodative convergence to accommodation ratio), peripheral refraction, pupil size, and treatment compliance. Thus, we plan to evaluate these factors as secondary and exploratory outcome measure items in the present study, as shown in Tables 1, 2, 3, and 4.

**Table 2:** Wearing experience questionnaire - 1

Activities		Content		
Parental myopia		0 person	1 person	2 persons
Clarity	Short distance	Good	Fair	Poor
	Intermediate vision distance	Good	Fair	Poor
	Long distance	Good	Fair	Poor
Time wearing spectacles (h/d)	Weekdays (Monday to Friday)			
	Weekends (Saturday to Sunday)			
Time spent at work	Near work (h)			
	Middle-distance work (h)			
Time spent on activities	Outdoor			
	Indoor			
Sleeping time				

Modified from Reference [10]

**Table 3:** Wearing experience questionnaire - 2

		Poorest	➞	Acceptable	➞	Fair	➞	➞	Good	➞	Excellent
1	Vision at a distance (clarity)	1	2	3	4	5	6	7	8	9	10
2	Vision stability at a distance	1	2	3	4	5	6	7	8	9	10
3	Clarity of vision for intermediate distances (e.g., computer, watching television)	1	2	3	4	5	6	7	8	9	10
4	Clarity of vision for near tasks (e.g., reading, using smartphone)	1	2	3	4	5	6	7	8	9	10
5	Vision stability at close range	1	2	3	4	5	6	7	8	9	10
6	Vision stability at a distance	1	2	3	4	5	6	7	8	9	10
7	Vision comfort	1	2	3	4	5	6	7	8	9	10
8	Vision outdoors	1	2	3	4	5	6	7	8	9	10
9	Ease of lens adaption	1	2	3	4	5	6	7	8	9	10
10	Overall performance	1	2	3	4	5	6	7	8	9	10

Modified from Reference [10]

**Table 4:** Wearing experience questionnaire - 3

		Do you have the following symptoms when you wear spectacles?									
		Never	➞	Seldom	➞	Sometimes	➞	➞	Often	➞	Always
1	Blurred vision at a long distance	1	2	3	4	5	6	7	8	9	10
2	Blurred vision at an intermediate distance (e.g., computer)	1	2	3	4	5	6	7	8	9	10
3	Blurred vision at a short distance (e.g., reading, smartphone)	1	2	3	4	5	6	7	8	9	10
4	Ghosting image	1	2	3	4	5	6	7	8	9	10
5	Unstable vision at a distance	1	2	3	4	5	6	7	8	9	10
6	Unstable vision at close range	1	2	3	4	5	6	7	8	9	10
7	Difficulty or slowness in refocusing your eye from one distance to other	1	2	3	4	5	6	7	8	9	10
8	Eyestrain	1	2	3	4	5	6	7	8	9	10
9	Double vision	1	2	3	4	5	6	7	8	9	10
10	Dizziness	1	2	3	4	5	6	7	8	9	10
11	Headache	1	2	3	4	5	6	7	8	9	10

Modified from Reference [10]

#### Cycloplegia protocol

All of the refraction and AL measurements will be obtained by a standard cycloplegia protocol. The recommended dosage for cycloplegic refraction is two drops of 1% tropicamide given 5 min separately. Cycloplegic refraction outcome measures will be obtained 30–45 min after the first drop of tropicamide is instilled, which ensures the maximal cycloplegic effect. Refraction will be measured with an open-field autorefractor (Shin-Nippon NVision-K 5001 autorefractor; Rexxam Co., Osaka, Japan). The AL will be measured by using an IOLMaster system (Carl Zeiss, Oberkochen, Germany). For SER and AL, five measurements will be obtained at each visit and then averaged for each eye for statistical analysis.

#### Follow-up examination and measurement schedule

All of the ophthalmic exanimation measures will be assessed at baseline, 1 month, 3 months, 6 months, and then every 6 months until 3 years after randomization. The differences in all of the mean values at each follow-up visit from baseline will be analyzed. Additionally, to avoid binocular interaction bias, only one eye will be randomly chosen for analysis of the study outcomes.

#### Training of the study staff

All staff from each site will be trained under the control of PKUPH, including in taking the standard measurements related to the primary, secondary, and exploratory outcomes; implementing the clinical trial protocol; managing data; and addressing key issues raised by participants. The training process is necessary for consistency, reproducibility, and repeatability. The standard training program will include but not be limited to obtaining BCVA, refraction, AL, binocular vision, corneal curvature, and peripheral refraction measurements; administering the questionnaire; and recording data. During the whole study, the SMO from PKUPH will play an internal quality control role to ensure the study is performed in a uniform manner at all study centers.

### Sample size calculation

Estimation of the sample size is based on the two following methods: statistical analysis and recommendations from review articles.

#### Statistical analysis

The two-sample *t* test for noninferiority statistical analysis was used for the sample size calculation. The noninferiority null hypothesis is that the refractive error, measured by SER, will be worse with DIMS lenses than with PALs for the treatment of myopia, and the rejection of this null hypothesis is powered to detect a noninferiority margin (Δ) of < 10% [15]. According to previously published articles, the 2-year difference in myopia progression with DIMS is 0.44 ± 0.09, whereas the difference in myopia progression with PALs is 0.20 ± 0.08 [10, 15, 16]. Thus, the mean difference between DIMS lenses and PALs is 0.22. Because the detected variation in myopia is ~ 0.25–0.5, we chose 0.5 as the SD factor. Other parameters used include a significance level of 0.05, 95% confidence interval (two-sided), 80% power, and 1:1 allocation. Based on these parameters, the estimated sample size for each group is 201 subjects. On the basis of our previous experience with clinical trials for treating myopia and other published data, we estimated the rate of subject loss to follow-up over 3 years to be ~ 40%. Considering these factors together, the estimated sample size for each group is 282.

#### IMI recommendation

The IMI summarized key issues in view of more than 170 peer-reviewed published articles on myopia control, and more than 85 multidisciplinary experts contributed to reports on clinical practice, basic research, and future directions [6–8, 15]. According to these IMI reports, key information about the sample size calculation is missing in published articles, and a reasonable number of subjects per group ranges from ~ 70 to 333 children over 2 to 3 years of follow-up [15]. In particular, the sample size per group for spectacles ranges from 125 to 333 [15].

Based on the expert consensus of the reviewed articles and the statistical calculation, a sample of 300 eligible children will be required in each arm of the trial. We do not plan to stratify any subgroups.

### Randomization and masking

The research coordinator will guide the participants to perform and finish all of the examinations, go through the results, and mask the groups to which the subjects belong. In addition, the coordinator will contact individuals before their follow-up visits.

The unmasked investigator (UMI) will be responsible for group allocation (i.e., allocating all of the children into either the DIMS lens group or PALs group through the sequence generation method [a random number table] at a 1:1 allocation ratio). In addition, the UMI will also be in charge of spectacle lens fitting, aftercare, performance assessments, data recording, and answering questions from participants and their parents/gradients.

The MI will be responsible for ophthalmic assessment and data measurements, blinded to the allocation, and not allowed to handle spectacle lenses throughout the study.

In addition, to avoid accidental unmasking, the spectacles will be kept by the UMI until the subjects finish the examination by the MI. Additionally, to avoid selection bias, allocation concealment will be ensured until the participants and their parents/guardians have been recruited into the clinical trial after the individuals finish all of the baseline examinations. During the clinical trials, neither the participants (together with their parents/guardians) nor the MI will be aware of group allocation.

### Data management and data analyses

Data from the two groups will be presented as the mean ± SD, except for the gender and patient number in each group, which will be presented as proportions. Data from a random eye will be used for data analysis according to a random table, considering the high correlation between the two eyes of the same participant. Baseline group data will be analyzed by using unpaired *t* tests. Repeated measures analysis of variance will be used to determine changes from baseline over time and between the two study groups. Bonferroni corrections will be used for post hoc comparisons. Correlations between changes will be calculated using Pearson’s correlation coefficient.

In the present study, all randomized participants will be included in the data analysis, regardless of protocol adherence. Missing data will not be included in the following analysis and will not be imputed from the time point of dropout. An interim analysis of the primary endpoint will be performed by an independent statistician when 50% of the participants have been allocated and have completed a 6-moth follow-up examination.

Once the subjects are enrolled, retention efforts will be addressed with participants and parents/guardians. Coordinators, study investigators, and examination staff will (1) provide periodic communication about the clinical trial and myopia control strategies for the subjects, (2) provide feedback regarding the eye care data of the subjects, and (3) provide reminders of the follow-up visit and final visit.

An internal data monitoring committee (DMC) will be established and will consist of ophthalmologists who are not involved in running the trial, statistical experts, and members of the ethics committee. The DMC chair will be Dr. Mu Shuang, the PKUPH ethics committee director. Data monitoring will be performed quarterly by the DMC, including monitoring for data completeness, safety information, adverse events, and so forth. No auditing will be performed through a professional organization. The integrity of the trial for each subject will be cross-checked between sites to ensure the appropriate allocation and completeness, accuracy, timeliness of data collection, and so forth.

In the present study, the main adverse event will likely be decreased visual clarity and discomfort after wearing glasses. Adverse events occurring after the dispensing of spectacle lenses will be recorded, and investigators will address the signs and symptoms of the subjects in a timely manner.

Participants may withdraw from the study for any reason at any time. In addition, the investigators may also withdraw participants from the study to protect their safety. All study-related information will be stored securely in locked file cabinets in the research laboratory at each study site. All of the records containing personal identifiers, such as names and informed consent, will be stored separately from data records identified by code number.

### Ethical approval and conduct

Ethical approval has been provided by Peking University People’s Hospital, and all amendments will be resubmitted to the ethics committee. Patient recruitment had not yet started at the time of manuscript submission.

## Discussion

### Necessity of the current study

The average age of myopia onset is 8 years in the United States and Singapore, whereas it is ~ 6–7 years in Asian countries other than Singapore [4, 17, 18]. In cases of myopia onset before the age of 8, there are not many alternative treatments, making spectacle lenses the main choice for parents and children [7, 8, 15]. Although the prominent theory of myopia control (SER and AL progression) hypothesizes that peripheral MD slows progression [9, 19, 20], evidence of the efficacy of various optical designs in children under the same inclusion criteria is lacking. Additionally, evidence has shown that the myopia control effects of plus defocus lenses are weaker and less consistent in human myopia clinical trials with spectacles [13, 14, 16, 21].

Additionally, MD spectacle lenses need to be adjusted regularly due to downward frame slippage to ensure that the child is looking through the near addition as much as possible for near vision while looking through the center for distant vision. When looking through the addition lenses, children undergo a special visual experience, which is not fully understood.

Thus, the current study aims to (1) investigate the myopia control effects of DIMS lenses and PALs through SER and AL changes in 600 children with early-onset myopia between 6 and 12 years old; (2) compare various indicators, including peripheral refraction, accommodation, contrast sensitivity, stereopsis, choroidal thickness, and wearing experience, over the course of 3 years; and (3) minimize bias through the cooperation of a multicenter and multiarea research group. The results will broaden understanding of whether and how much the different MD designs retard myopia progression.

### Rationale for the study design

Myopia is a progressive eye disease that has been reported to remain stable for ~ 16 years [4]. Because myopia control interventions will be applied for multiple years through the time myopia is progressing, it is important for clinical trials to evaluate efficacy over a long period to ensure continued efficacy beyond any initial treatment effect [15]. Several clinical trials have shown evidence of diminishing efficacy beyond the first year, with no continuous myopia progression control after 1 year of treatment during the 2 subsequent years [6, 15]. This phenomenon could lead to incorrect decisions in clinical consensus. Thus, as recommended by the IMI, 3 years was chosen as the follow-up duration of the present clinical trial assessing the treatment efficacy of different MD spectacle lenses [15, 16].

An appropriate control group is a key factor for evaluating efficacy in a clinical trial. Although placebo or SV spectacles are recommended for the control group, we did not choose to include either kind of control group in the present study. Instead, we included a large number of subjects (*n* = 300 in each group) to directly compare the myopia control effect of spectacles with DIMS lenses and PALS in 6- to 12-year-olds. The reasons behind not choosing SV spectacles and placebo control are as follows:

1. Once a myopic child has been identified, an appropriate treatment strategy to manage myopia progression must be selected; thus, it is not possible to establish a placebo control.
2. SV spectacles have been proven to have little or no effect on myopia progression.
3. Patients and their guardians rarely agree to accept SV lenses.
4. SV spectacles are not recommended as a first-line treatment strategy based on the consensus of the *Chinese Journal of Optometry Ophthalmology and Visual Science*.

Considering the risk-to-benefit assessment for the patients in the long-term clinical trial, we will test only the spectacle lenses that have already been found effective in myopia control.

### Principle of outcome selection

Visual function has many aspects, so it is recommended to be included in clinical trials evaluating myopia control [15]. The most common primary outcome measure in myopia control studies is refractive error, which is directly related to the tested treatment efficacy. To ensure maximal consistency of the measured results at each research center, we specified a standard method for cycloplegia (refer to the “Methods” section above).

In the present study, we chose subjective refraction measured by phoropter as the main primary outcome, not the objective refraction measured by autorefraction. For clinical application and clinical trial assessment [10], cycloplegic subjective refraction is recommended as an endpoint measurement [22], especially for the patient who has optical aberrations, such as astigmatism.

To minimize evaluation bias and help with data interpretation, several indicators related to changes in refractive error and myopia progression will also be measured, including AL, corneal curvature, peripheral refraction, parental myopia status, environmental influences, and education insensitivity. In addition, the self-report questionnaire will be used to evaluate compliance and wearing experience [23].

As mentioned before, the underlying principle through which MD can slow myopia progression is that it provides blurry images in front of the retina when objects are viewed at close range. MD may induce unexpected effects on vision, including aspects such as contrast sensitivity, stereopsis, accommodation, and convergence. Therefore, in addition to regular examination items, all of the aforementioned items will be measured in the current study.

MD has been proven to reduce myopia progression and axial elongation and has gained great interest in the context of preventive treatment for myopia, with few adverse effects seen in early childhood. The purpose of this study is to investigate the myopia control effects of two types of MD spectacle lenses over 3 years of follow-up in 6- to 12-year-old schoolchildren. The findings from the present study are proposed as a resource to inform future clinical practices.

### Trial status

Ethical approval has been obtained from Peking University People’s Hospital (protocol version number 2019PHA049-001, V1.0; dated 2019 Sept 9). Participant recruitment has not yet begun as of this submission. The clinical trial is started to recruit participants from 30 October 2019, and the approximate date when recruitment will be completed is 30 October 2020.

## Supplementary information

**Additional file 1.** Standard Protocol Items: Recommendations for Interventional Trials (SPIRIT) 2013 checklist: recommended items to address in a clinical trial protocol and related documents.

## Data Availability

Not applicable. Data sharing is not applicable to this article, because no datasets have been generated or analyzed in the current study. After the clinical trial is finished, the original data will be uploaded to the ResMan Primitive Data Sharing Platform (IPD Sharing Platform) of the China Clinical Trials Registry, http://www.chictr.org.cn/index.aspx.

## Abbreviations

AL: Axial length
BCVA: Best corrected visual acuity
ChiCTR: Chinese Clinical Trial Registry
DIMS: Defocus incorporated multiple segments
DMC: Data monitoring committee
IMI: Interventions Myopia Institute
MD: Myopic defocus
MI: Masked investigator
PALs: Apollo progressive addition lenses
PKUPH: Peking University People’s Hospital
SER: Spherical equivalent refraction
SMO: Site management organization
SV: Single vision
UMI: Unmasked investigator
